# Transient Arterial Hypertension Induced by Gonadotropin-Releasing Hormone Agonist Treatment for Central Precocious Puberty

**DOI:** 10.3389/fped.2019.00074

**Published:** 2019-03-19

**Authors:** Loukia Sifaki, Francois Cachat, Gerald Theintz, Hassib Chehade

**Affiliations:** ^1^Department of Woman-Mother-Child, Lausanne University Hospital, Lausanne, Switzerland; ^2^Private Practice for Paediatric Endocrinology, Lausanne, Switzerland

**Keywords:** arterial hypertension, central precocious puberty, Gonadotrophin releasing hormone agonist, triptorelin, child—age

## Abstract

**Background:** Gonadotropin-releasing hormone agonists (GnRHa) are a safe and effective treatment for precocious puberty. Triptorelin is one of the long lasting GnRHa, which reversibly suppresses the pituitary-gonadal axis. Triptorelin-induced hypertension (HTN) has rarely been reported in the literature.

**Clinical Case/Methods:** We report a 10-year-old girl with central precocious puberty who, during treatment with triptorelin, developed an asymptomatic stage II HTN. Initial workup showed no renal, thyroid, or electrolytes abnormalities. The renal ultrasound showed no parenchymal disease and no increased renal resistance index suggestive of a renal artery stenosis. Echocardiography and ocular fundoscopy were normal. HTN (stage II) was confirmed with ambulatory blood pressure monitoring (ABPM). After extensive literature review, we found 3 other cases of HTN secondary to GnRHa, improving with endocrine treatment cessation. Therefore, antihypertensive treatment was not started immediately in our patient. Indeed, after completion of her treatment with triptorelin, we observed a complete normalization of her blood pressure (confirmed with ABPM) without any medication.

**Conclusion:** Concomitantly to GnRHa treatment, our patient developed HTN, which completely subsided after stopping triptorelin. The complete normalization of her blood pressure, together with a negative workup for HTN strongly speaks for a causal effect of her endocrine treatment. In this setting, estrogen depletion might play a role, although this remains debated.

## Introduction

The incidence of central precocious puberty (CPP) in children is ~1/5,000–1/10,000 with a female to male ratio of 20:1 (1). Gonadotropin-releasing hormone agonists (GnRHa) are the treatment of choice for CPP. GnRHa administration results in an initial stimulation of pituitary gonadotropin secretion followed by a complete and reversible suppression of the pituitary-gonadal axis. Triptorelin, a long-lasting GnRHa, decreases testosterone levels in boys and estrogen levels in girls. Triptorelin is a widely used drug with few reported adverse effects, such as erythema, bruising, pain, sterile abscesses, menopausal-like symptoms, and anaphylaxis (2, 3). So far secondary arterial hypertension (HTN) has been reported in only 3 patients with CPP (4–6).

We here report a new case of a 10-year-old girl with CPP who developed a stage II HTN during triptorelin treatment, followed by a complete normalization of her blood pressure after triptorelin discontinuation, without any pharmacological anti-hypertensive treatment.

## Case Report

An otherwise healthy 10-year-old girl was referred to the pediatric nephrology unit for further investigation and treatment of an asymptomatic stage II HTN (defined as ≥95th percentile + 12 mm Hg). The girl was born at term, after an uneventful pregnancy (birth weight 3,650 g). There were no reported post-natal complications. Family history was remarkable for treated HTN in her father since the age of 35 years. At the age of 7 years, our patient was referred to a pediatric endocrinologist for further investigation of bilateral breast development and pubic hair growth, which started 6 months earlier. On clinical examination, weight was 34 kg (+4.61 Standard Deviation Score, SDS), height 140 cm (+4.42 SDS), body mass index 17.35 kg/m^2^ (+1.44 SDS) and the latest annual growth was 12.6 cm/year (+7.77 SDS). Blood pressure was 99/66 mmHg (<90th percentile). She had a Tanner stage 3 breast development, a Tanner stage 3 pubic hair and a moderate axillary hair. Bone age (Greulich-Pyle) was already advanced at first visit (8 years for a chronological age of 7). Pelvic echography displayed an enlarged uterus for age (uterine volume of 5.5 ml). The right ovary was also enlarged for age (2.6 ml) with some follicles. The left ovary could not be seen due to overlying bowel gas. The magnetic resonance imaging of hypothalamus-pituitary axis (performed after the Luteinizing Hormone Releasing Hormone (LHRH) stimulation test) was normal. Estradiol level at first visit was 19 pmol/l. Central isosexual precocious puberty was confirmed with a positive LHRH stimulation test: LH peak 17.6 mU/l and Follicle Stimulation Hormone (FSH) peak 11.8 mU/l. To protect the child from a distress of the dissociation induced by a mature (pubertal) body in a psychologically and emotionally immature person, encompassing obvious differences in school and among peers, decision was taken to treat her. The patient received 3.75 mg (one set) at the onset of therapy. A second set was injected 14 days later, following strictly the recommendations provided by the manufacturer (Ferring). No allergic reaction was observed. However, an increased blood pressure was noticed after 2 months treatment (121/80 mm Hg). Blood pressure remained elevated throughout the period of treatment leading to stop it at 9.5 years, whereas the goal was to continue until 10 years.

She was then referred to the nephrology clinic for an asymptomatic high blood pressure (139/85 mmHg), confirmed with an ambulatory blood pressure monitoring (ABPM). Mean day BP was 140/96 mmHg, mean night BP was 121/76 mmHg, and systolic blood pressure load was 62.5%. Clinical exam was normal and the initial workup showed no renal, thyroid, or electrolytes abnormalities. There was no proteinuria. The renal ultrasound showed no parenchymal disease and no increased renal resistance index suggestive of a renal artery stenosis. Echocardiography and fundoscopy were normal. After reading the above-mentioned case reports, we purposely postponed any antihypertensive treatment, hoping for a spontaneous normalization of her blood pressure. Indeed, 6 weeks after stopping GnRHa treatment, the girl had a normal office BP, and a normal ABPM with a mean day and night BP values of 115/76 and 108/70 mmHg, respectively.

## Discussion

GnRHa represent the treatment of choice in children with CPP. HTN occurrence during treatment has rarely been reported. So far, only three cases of children with CPP and secondary HTN during treatment with GnRHa have been published. Calcaterra et al. (4) recently described a 7-year-old girl with Triptorelin-treated CPP, who developed reversible HTN with secondary concentric left ventricular hypertrophy, requiring transient antihypertensive therapy. The authors suggested a causal relationship between GnRHa treatment and HTN (4). Siomou et al. (5) also described a case of a 10-year-old girl with a Williams-Beuren syndrome and CPP who developed HTN with Triptorelin treatment. In that case, blood pressure totally normalized, without any anti-hypertensive medication once GnRHa was discontinued (5), similar to our case. In both cases, re-exposition to the offending drug was not performed. Finally, Palma et al. also very recently report a girl with CPP and arterial HTN during treatment with Triptorelin, HTN that subsided once Triptorelin was interrupted (6).

Arterial HTN has been observed in other clinical settings as well. Klink et al. report three adolescent girls with gender dysphoria during treatment with the GnRHa Triptorelin (7). Re-exposure to the offending drug in on patient strongly points toward a causal relationship between Triptorelin and HTN. Also, in adult patients, especially men with prostate cancer, GnRHa has been associated with changes in body composition, obesity, insulin resistance, and hypertension (8). The mechanisms leading to arterial HTN are still unclear. Several authors have investigated the pathophysiological mechanisms that underlie the occurrence of HTN due to GnRHa therapy in animal models. Acs et al. (9) showed in a rat model that triptorelin treatment decreases estradiol levels and the passive diameter of the saphenous artery, which were completely restored after estrogen replacement therapy. These results suggest that the background mechanisms for the increased incidence of HTN during GnRHa treatment might be secondary to estrogen depletion (9). Várbíró et al. (10) in a model of Triptorelin-ovariectomised rats, report significant biomechanical changes, and alteration in the venous distensibility. These abnormalities were completely reversed with estradiol and medroxyprogesterone acetate hormone replacement (10). Although estrogen depletion may play a role in the pathogenesis of Triptorelin-induced HTN, this should be further investigated. Finally, whereas the blood pressure of a growing child is usually matched to normal values for age and sex, it should rather be matched to height or weight age as one of the hallmark of central precocious puberty is to induce a high growth velocity and therefore relatively tall stature with increased weight gain. In an observational study (11), blood pressure of children with CPP was significantly increased but generally appropriate for height age or weight age.

In our case report, the presence of a normal BP before initiation of Triptorelin and the complete normalization of the blood pressure after stopping it, points toward a causal effect of the GnRHa treatment. Given the sequence of pharmacological interventions and the current literature data, we speculate that the HTN was secondary to GnRHa treatment. We felt it unnecessary and unethical to re-challenge the patient with Triptorelin. On the basis of our case, 3 previously reported cases and other supporting literature data, we recommend monitoring the blood pressure in every child receiving GnRHa treatment, and in particular Triptorelin.

We obtained parental consent from the parents of the patient for the publication of this case report.

## Ethics Statement

This is the description of a rare adverse event of an approved drug, in an approved condition.

## Author Contributions

LS, FC, GT, and HC contributed to the care of the patient, the redaction of the case report and the review of the literature, and take full responsibility of the manuscript.

### Conflict of Interest Statement

The authors declare that the research was conducted in the absence of any commercial or financial relationships that could be construed as a potential conflict of interest.
